# Sensitivity of markers of DNA stability and DNA repair activity to folate supplementation in healthy volunteers

**DOI:** 10.1038/sj.bjc.6603197

**Published:** 2006-05-30

**Authors:** G P Basten, S J Duthie, L Pirie, N Vaughan, M H Hill, H J Powers

**Affiliations:** 1 Human Nutrition Unit, Division of Clinical Sciences (North), The University of Sheffield, Northern General Hospital, Sheffield S5 7AU, UK; 2 Rowett Research Institute, Aberdeen AB21 9SB, UK

**Keywords:** folic acid, health, uracil misincorporation, DNA damage

## Abstract

We have previously reported that supplementation with folic acid (1.2 mg day^−1^ for 12 week) elicited a significant improvement in the folate status of 61 healthy volunteers. We have examined effects of this supplement on markers of genomic stability. Little is known about the effect of folate supplementation on DNA stability in a cohort, which is not folate deficient. Preintervention, there was a significant inverse association between uracil misincorporation in lymphocyte DNA and red cell folate (*P*<0.05). In contrast, there were no associations between folate status and DNA strand breakage, global DNA methylation or DNA base excision repair (measured as the capacity of the lymphocyte extract to repair 8-oxoGua *ex vivo*). Folate supplementation elicited a significant reduction in uracil misincorporation (*P*<0.05), while DNA strand breakage and global DNA methylation remained unchanged. Increasing folate status significantly decreased the base excision repair capacity in those volunteers with the lowest preintervention folate status (*P*<0.05). Uracil misincorporation was more sensitive to changes in folate status than other measures of DNA stability and therefore could be considered a specific and functional marker of folate status, which may also be relevant to cancer risk in healthy people.

Epidemiological studies have consistently revealed an association between the consumption of fruit and vegetables and reduced incidence of cancers at various sites. Although the active dietary components have not been identified, folate has received a significant amount of attention. A relative deficiency of folate has been associated with precancerous lesions or cancer of the breast, large bowel, cervix, oesophagus, lung and pancreas (Jaskiewicz *et al*, 1988; Jaskiewicz, 1989; Van Eenwyk *et al*, 1990; Lashner, 1993; Duthie, 1999; Giovannucci, 2002; Zhang *et al*, 2003).

There are currently thought to be two main routes whereby folate deficiency might increase malignant transformation, both reflecting the importance of folate in 1-C metabolism. The first deals with the role of folate in normal DNA synthesis and DNA repair. Folate is essential for the *de novo* synthesis of purines (A and G) and pyrimidines (T and C), which are required during the replication and repair of DNA. Folate in the form 5,10 methylenetetrahydrofolate is essential for the conversion of deoxyuridylate to thymidylate, but when folate is limiting, uracil is misincorporated into DNA which may ultimately lead to DNA double-strand breakage, chromosomal damage and malignant transformation (Reidy, 1988; Blount and Ames, 1994). Increased uracil misincorporation into DNA has been reported in various *in vitro* systems under a folate restricted environment (Libbus *et al*, 1990; Wickramasinghe and Fida, 1993; Branda *et al*, 1994; James *et al*, 1994; Duthie and Hawdon, 1998; Melnyk *et al*, 1999). The negative effect of folate deficiency on genomic stability is also supported by studies in animals fed folate-depleted diets (James and Yin, 1998; Duthie *et al*, 2000a). Evidence for the effects of folate status on uracil misincorporation in humans is scarce but there is some evidence that uracil misincorporation into lymphocyte and bone marrow cell DNA is increased in folate deficiency (Wickramasinghe and Fida, 1994; Blount *et al*, 1997). In addition to altered thymidine synthesis, purine biosynthesis is negatively affected by low folate (10-formyltetrahydrofolate), thereby limiting the availability of nucleotides for DNA synthesis and repair, which will also have consequences for genomic stability. Repair of oxidative DNA damage is compromised in folate deficient cultured human and rat colonocytes *ex vivo* (Duthie *et al*, 2000b, 2000c). It remains to be established whether increasing folate availability can improve DNA repair activity.

The second route whereby folate status may influence cancer risk is via its function in DNA methylation and gene expression. Folate, in the form 5-methyltetrahydrofolate (5-MeTHF), acts as methyl donor in the remethylation of homocysteine to methionine, which in turn is converted to *S*-adenosylmethionine (SAM). *S*-adenosylmethionine is the direct methyl donor in the methylation of DNA. Folate deficiency, by limiting cellular SAM levels, may induce global DNA hypomethylation and increase the risk of malignant transformation. While it is generally accepted that extreme methyl depletion alters DNA methylation and increases cancer risk in rodents, the effect of folate deficiency alone on cytosine methylation is not so clear. Hepatic DNA is hypomethylated in rats fed a folate depleted diet for 4 weeks (Balaghi and Wagner, 1993), while folate deficiency results in the preferential hypomethylation of exons 6 and 7 of p53 in rat colon (Kim *et al*, 1995). Conversely, other studies have described no effect of moderate folate deficiency on DNA hypomethylation (global or site-specific) in rat liver or colon (Kim *et al*, 1995; Duthie *et al*, 2000c; Sohn *et al*, 2003). The data suggesting a role for folate in modulating DNA methylation in humans is equally inconsistent. Global DNA hypomethylation has been reported to be increased in lymphocytes isolated from elderly women with folate deficiency (Jacob *et al*, 1998; Rampersaud *et al*, 2000). In contrast, global DNA methylation was found not to be influenced by folate status in a study of younger subjects (Fenech *et al*, 1998). Little is known about the effect of folate supplementation on global DNA methylation in healthy nonfolate deficient human volunteers.

The enzyme methylenetetrahydrofolate reductase (MTHFR; EC 1.5.1.20), responsible for the conversion of 5,10-methyleneTHF to 5-MeTHF, is polymorphic, with 5–15% of populations homozygous for the variant C677T, which decreases the efficiency of the enzyme (Frosst *et al*, 1995). The associated decrease in 5-MeTHF might be expected to lead to global DNA hypomethylation and an increased risk of cancer. However, this mutation might also be expected to lead to an increase in 5,10-methyleneTHF (Bagley and Selhub, 1998), which should drive increased thymidine production for DNA synthesis and repair. Homozygosity for C677T is associated with a reduced risk of colorectal cancer in subjects with good folate status (Ma *et al*, 1997), suggesting that the impact that folate status has on genomic stability and repair may be significant in the process of malignant transformation and indicating that uracil misincorporation may indeed be an important early cancer biomarker in humans. The importance of this genotype to cancer risk at other sites is less clear.

There is no evidence in the literature for effects of increasing folate status on DNA stability (uracil misincorporation, global strand breakage, DNA base excision repair activity or global DNA methylation) in healthy nonfolate deficient volunteers, even though this group is most commonly used to report epidemiological links between diet and cancer. In the present study, we evaluated the effects of improving folate status on markers of DNA stability in lymphocytes.

## MATERIALS AND METHODS

### Volunteer selection and sample size

Volunteers were recruited to the study as previously reported (Basten *et al*, 2004). Briefly, a randomised double-blind placebo-controlled, folic acid intervention trial was carried out in healthy men and women (20–60 years). Volunteers who were pregnant or planning a pregnancy, receiving medical care, taking methotrexate, antiepileptic drugs or vitamin supplements, who were smokers or who had a red cell folate (RCF) concentration less than 200 nmol l^−1^ (folate deficient; UK Department of Health) were excluded. DNA methylation acceptance in isolated lymphocytes from human volunteers decreased by 38% when folate intake increased from 100 to 516 *μ*g day^−1^ (Jacob *et al*, 1998). To detect a similar improvement in DNA methylation in response to folate supplementation with a power of 95% and a probability of 5% would require 28 subjects per group. We aimed to recruit 30 per group to allow for drop-outs from the study. The study was reviewed and approved by the North Sheffield Ethics Office of the Sheffield Teaching Hospitals NHS Trust, UK. Written informed consent was obtained from all subjects.

### Intervention dose

Volunteers were randomised to receive either 1.2 mg folic acid (pteroylglutamic acid) or glucose placebo, daily for 12 weeks. The folate dose represents a significantly higher dose than the current UK and US recommended nutrient intake of 200 *μ*g for adults, but is considerably lower than the pharmacological doses used in many clinical trials (Areekul *et al*, 1980; Butterworth *et al*, 1992; McGregor *et al*, 2000; Kim *et al*, 2001; Cafolla *et al*, 2002). Intervention capsules were manufactured by the Clinical Directorate of Pharmacy Services, Sheffield Teaching Hospitals NHS Trust, UK.

### Specimen collection

As described previously (Basten *et al*, 2004) subjects were initially screened for RCF status by donating a 500 *μ*l sample of whole blood, obtained from the finger tip using a pro-lancet device (Roche, UK) into an EDTA coated tube (Startsedt, UK). In all, 65 subjects with RCF between 250 and 650 nmol l^−1^ were recruited to the intervention trial. This range represents a less than average folate status for the adult UK population, while excluding folate deficient volunteers. Volunteers donated a 20 ml sample of 16 h fasted blood into EDTA-containing evacuated tubes at week 0, and following 12 weeks of supplementation. Lymphocytes were prepared from fresh whole blood collected at week 0 and week 12 with and specifically stored in duplicate for the later measurement of DNA stability markers.

### Lymphocyte preparation to measure uracil misincorporation, DNA strand breakage, DNA base excision repair and global DNA methylation

Human lymphocytes were isolated as described previously (Basten *et al*, 2004). In brief, whole blood was centrifuged at 1500 g at 4°C for 15 min. The ‘buffy coat’ layer was removed, re-suspended in RPMI media (Gibco, UK), and layered onto an equal volume of lymphoprep gradient solution (Robbins Scientific, UK). Following centrifugation at 700 g, 25°C for 30 min, the cells were washed three times using RPMI (25°C, 15 min, 700 g), and counted using either a haemocytometer or automated cell counter (Coulter, Fullerton, CA, USA). For the measurement of uracil misincorporation, DNA strand breakage and DNA methylation 500 *μ*l of lymphocytes were cryopreserved in 90% FCS/10% DMSO at a final cell density of 3 × 10^6^ ml^−1^ and stored at –80°C. For the measurement of base excision repair capacity the final cell pellet was resuspended in extraction buffer at a cell density of 10^7^/100 *μ*l, snap frozen in liquid nitrogen and stored at −80°C.

### Lymphocyte uracil misincorporation and DNA strand breakage status

DNA instability (strand breaks and misincorporated uracil) was measured in lymphocytes isolated by single cell gel electrophoresis as described previously (Duthie and McMillan, 1997; Narayanan *et al*, 2001). A total of 100 comet images from each gel (with duplicate gels per subject) were classified according to the intensity of fluorescence in the comet tail representing damaged cells. Images were scored randomly across each gel and the experimenter was blind to the intervention. DNA strand breakage was estimated based only on the score obtained from buffer-treated gels. Misincorporated uracil was measured by subtracting the visual score obtained from buffer-treated gels (endogenous strand breakage) from the score obtained after incubation with uracil DNA glycosylase (strand breakage due to the excision of uracil). This method of classification has been validated extensively by computerised image analysis (Komet 3.0, Kinetic Imaging Ltd, Liverpool, UK) (Duthie and Hawdon, 1998). Quality control was maintained through the use of a standard lymphocyte lysate, which was independently validated and quantified through internal quality control procedures. All measurements were made blind to code and quality control standard. The coefficient of variation for the assay was below 13%.

### Base excision repair capacity (number of base incisions)

Base excision repair capacity in supplemented or unsupplemented volunteers was assessed as the ability of cell-free extract (prepared from isolated lymphocytes as above) to excise 8-oxo-7,8-dihydroguanine (8-oxoGua) from substrate DNA isolated from CHO cells treated with either PBS or RO19-8022 and light (Collins *et al*, 2001). Rate of excision of 8-oxoGua was detected as an increase in DNA strand breaks using conventional SCGE as described previously (Collins, 2004). DNA incision was calculated as the net increase in DNA strand breakage in RO-treated cells incubated with lymphocyte lysate from 0 to 20 min after subtracting equivalent scores from PBS-treated cells incubated with extract. In this way, any nonspecific incision activity due to incubation at 37°C with extract alone was accounted for. The coefficient of variation across the study was below 22%.

### Lymphocyte DNA methylation status

DNA was isolated from lymphocytes using a Qiagen DNA Blood Mini kit. Total genomic DNA methylation was determined by measuring incorporation of methyl groups from ^3^H-labeled *S*-adenosyl-L-methionine at specific cytosine residues using the bacterial enzyme Sss1 methylase. DNA methylation status is inversely related to the degree of radioactive incorporation, that is the lower the methylation of the DNA the higher the disintegrations per minute (DPM) (Balaghi and Wagner, 1993). Standard lymphocyte DNA was coanalysed with each group of samples. The coefficient of variation for the assay was 6.9%.

### Measurement of whole blood SAM by HPLC-UV

For the measurement of SAM concentration, 100 *μ*l of whole blood were deproteinised in 900 *μ*l of 6% perchloric acid (w/v) and stored at −80°C. *S*-adenosylmethionine was measured using a modified HPLC assay based on published methods (Bottigleri, 1990; Loehrer *et al*, 1996). SAH is present in blood in much lower concentrations than SAM and the method employed was not adequate to quantify SAH with the desired degree of precision. A Gilson G715 HPLC with autoinjector (G234 was used, with samples held at a temperature of 4°C, one pump (G305) and a UV detector (G116), on a Nucleosil 100 C8 column (250 × 4.6 mm) (Supelco, UK) with a mobile phase of 0.1 M sodium acetate and 2% acetonitrile (pH 4.5). The flow rate was 1 ml min^−1^, with detection at 254 nm (0.01 AUFS) and using an injection volume of 50 *μ*l. Inter-batch coefficient of variation was 8.2%

### MTHFR Genotype

A whole blood aliquot was stored at −80°C for the later determination of MTHFR genotype. MTHFR genotype was determined using a modification of published methods (Frosst *et al*, 1995; Clark *et al*, 1998). The DNA was isolated from whole blood according the QIAamp DNA Mini Kit protocol (Qiagen, UK) and the DNA quantified using a Picogreen dye (Molecular Probes, Netherlands). MTHFR genotype was determined by polymerase chain reaction (PCR) using primers: sense 5′-TGAAGGAGAAGGTGTCTGCGGGA-3′, antisense: 5′-AGGACGGTGCGGTGAGAGTG-3′. Predetermined MTHFR genotype control DNA was a kind gift from Dr P Guthrie, North Trent Molecular Genetics Laboratory, Sheffield Children's Hospital, UK.

### Data analysis

Statistical analyses were performed with SPSS software, version 12.0 (SPSS Inc., Chicago). Correlations between variables were evaluated with the use of Spearman's rank-order coefficient of correlation. Kruskal–Wallis nonparametric independent samples test was used to evaluate the effect of intervention, using percentage change from baseline as test variable against intervention (folate or placebo) as grouping variable. All tests were two-tailed and were considered significant when *P*<0.05.

## RESULTS

### Preintervention biochemical measurements

A total of 61 volunteers completed the intervention. The supplement group consisted of 15 men and 15 women, mean ages 42 year. (±9 year) and the placebo group consisted of 15 men and 16 women, 40 year. (±8 year). We have previously reported a significant effect of folic acid supplementation on measures of folate status and function (Basten *et al*, 2004). This report examines associated effects on measures of DNA stability and repair.

Plasma concentration of SAM, and measures of DNA stability and repair are shown in Table 1. Values are shown for samples collected pre- and post intervention. Although we specifically recruited self-reported nonsmokers plasma cotinine concentrations suggested that 10 subjects had been exposed to sufficient tobacco smoke to elevate plasma cotinine. However, plasma cotinine was not significantly associated with any measure of folate status or DNA stability and appears not to be a confounder in this study. There was no significant effect of MTHFR C677T genotype on any of the preintervention measures of DNA stability. This should be expected, however, as the recruitment was not based on genotype, and because of this the numbers with the TT variant were small (supplement *n*=3, placebo *n*=4). There was a significant negative association between preintervention uracil misincorporation and RCF (*P*<0.01 *r*=−0.5). There was, however, no significant association between any marker of preintervention folate status and baseline DNA methylation, DNA strand breakage, or the base excision repair capacity.

### Effects of folic acid supplementation

As previously reported, folate supplementation elicited a significant increase in plasma 5-MeTHF, red cell folate and lymphocyte total folate compared with the control group (*P*<0.01) (Basten *et al*, 2004). We now show that folate supplementation significantly increased whole blood SAM (*P*<0.01). The increased concentration of SAM was closely associated with the increase in red cell folate and plasma 5-MeTHF concentrations (*P*<0.01). Similarly, supplementation elicited a significant (*P*<0.01) reduction in plasma homocysteine compared with the control group (Basten *et al*, 2004).

**Figure 1:**
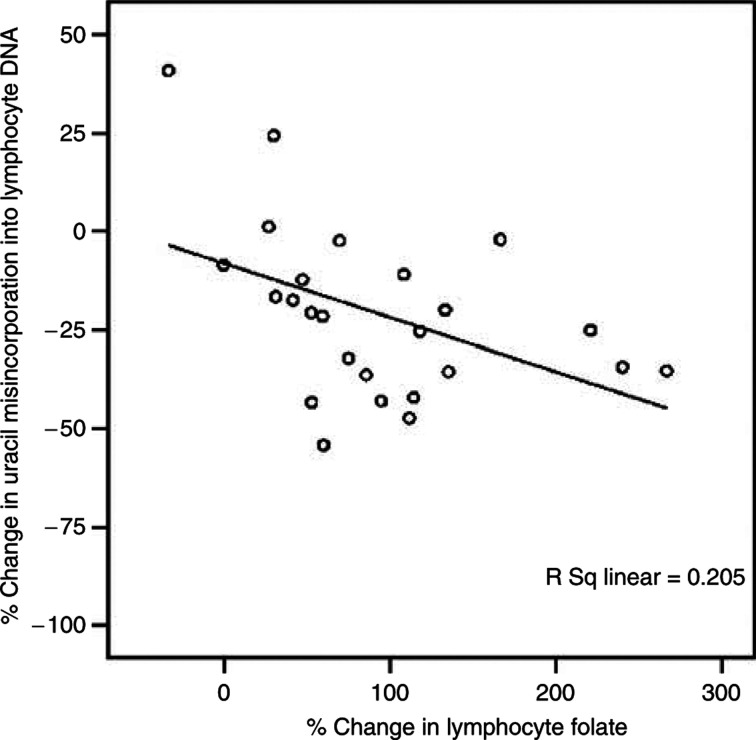
Association between lymphocyte folate and lymphocyte DNA uracil misincorporation.

A significant decrease in uracil misincorporation (*P*<0.05) was observed in the folate-supplemented group compared with control. There was a small decrease in uracil misincorporation observed in the placebo group, but this was not significant. Moreover, there was a strong correlation between the magnitude of the increase in lymphocyte total folate and reduction in lymphocyte DNA uracil misincorporation (Figure 1; *P*<0.01 *r*=−0.49). There was also a significant negative association between changes in uracil misincorporation and changes in plasma 5-methyl THF (*P*<0.01, *r*=−0.48) and RCF (*P*<0.01, *r*=−0.46). Uracil misincorporation was examined according to preintervention quartiles of RCF status. The effect of folate supplementation was even more pronounced in the lowest quartile of preintervention RCF; although the numbers are small (*n*=7) the observation is still of interest.

Conversely, DNA strand breakage and global DNA methylation were unaffected by supplementation and no associations were observed between these markers of DNA stability and folate status either when analysed as groups or when stratified by RCF status.

**Figure 2:**
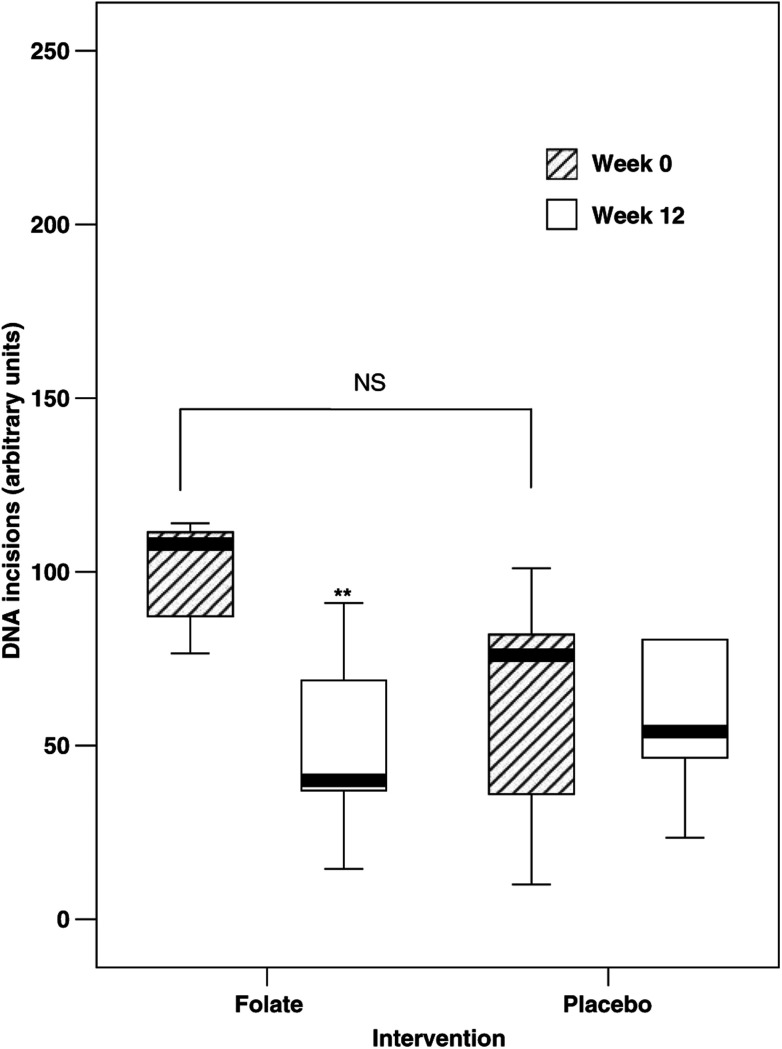
Effect of folate supplementation on DNA incision (lowest RCF quartile).

Folate supplementation had no effect on the capacity of the lymphocyte lysate to repair 8-oxoGua *ex vivo* when using the data from the whole study. The capacity of the lymphocyte lysate to repair 8-oxoGua *ex vivo* was then examined according to preintervention quartiles of RCF status. In the lowest quartile, folate supplementation elicited a significant decrease in the number of base incisions compared with control (*P*<0.05) (Figure 2).

## DISCUSSION

Low intake of folic acid has been implicated in the development of certain epithelial cell cancers (Jaskiewicz *et al*, 1988; Jaskiewicz, 1989; Van Eenwyk *et al*, 1990; Lashner, 1993; Giovannucci, 2002; Zhang *et al*, 2003). Folate deficiency has been hypothesised to increase cancer risk either by perturbing DNA synthesis and repair (Reidy, 1988; Blount and Ames, 1994) or by inducing DNA hypomethylation and negatively affecting gene expression (Balaghi and Wagner, 1993). However, putative biomarkers of folate status related to cancer risk have not been adequately explored in healthy volunteers. In order for a marker of folate status to be useful as a biomarker of cancer risk, it must be sensitive to changes in folate intake in healthy people. We conducted a randomised, double blind, placebo-controlled intervention trial in healthy volunteers to investigate whether putative biomarkers of DNA stability were sensitive to a 12-week supplementation with 1.2 mg folic acid. We have previously reported significant improvement in measures of folate status in plasma, red blood cells and lymphocytes, and a reduction in plasma homocysteine (Basten *et al*, 2004). The present report reveals that folate supplementation increases whole blood SAM and elicits a significant effect on some measures of DNA stability and repair.

Uracil misincorporation into lymphocyte DNA was negatively associated with measures of folate status at baseline (RCF) and postintervention (RCF, lymphocyte folate, plasma folate). Of particular interest, however, is that in this study, folate supplementation significantly decreased misincorporated uracil in lymphocyte DNA but did not alter global DNA strand breakage or DNA methylation, confirming the importance of folate in the synthesis of thymidine. This reduction in uracil following folate supplementation was most pronounced in those with the poorest folate status at baseline and while the number of subjects is small, the observation indicates that an important functional deficit is readily corrected by folic acid supplementation. These results are consistent with effects reported in two patient groups. There have been two other reports of effects of folate status on uracil incorporation in DNA. Blount *et al* (1997) observed an effect of high-dose folic acid in splenectomised patients. Wickramasinghe and Fida (1994) reported increased uracil incorporation in DNA in patients with megaloblastic anaemia compared to three healthy control subjects. However, we believe this is the first study to show that folic acid supplementation in healthy human volunteers who are not folate deficient, can decrease uracil misincorporation.

Uracil in DNA is also reported to decrease in human lymphocytes and colonocytes cultured with increasing concentrations of folic acid (Wickramasinghe and Fida, 1994; Duthie *et al*, 2000b). Conversely rats fed a folate-deficient diet for 10 weeks exhibited elevated uracil misincorporation into lymphocyte DNA (Duthie *et al*, 2000a). Additional restriction of dietary methionine and choline, both dependent upon folate for their metabolism, increased DNA strand breakage in blood cells but had no effect on uracil misincorporation, confirming the specificity and dependency of this biomarker on folate status.

To our knowledge, this is the first human study to determine the effects of folate supplementation on DNA base excision repair capacity. DNA repair is crucial in maintaining genomic stability and compromised DNA repair is associated with increased risk of malignancy (Reidy, 1988; Blount and Ames, 1994). Given the profound influence that folate status has on DNA metabolism, and preliminary indications that folate deficiency can negatively influence repair of oxidative DNA damage *in vitro* (James and Yin, 1998; Duthie *et al*, 2000a), it might be expected that DNA base excision repair capacity could be improved by increasing folate intake. Despite significant increases in blood and lymphocyte folate levels, supplementation in the intervention group as a whole did not alter initiation of DNA base excision repair. This suggests that DNA repair activity might not be altered by increasing folate intakes in people with adequate folate status as determined by conventional measures. However, in the lowest preintervention RCF quartile, folate supplementation significantly decreased DNA repair incision activity compared to placebo. A definitive explanation for this surprising observation is lacking at this time, although it may simply reflect a downregulation in repair activity due to increased provision of nucleosides following folate supplementation in this relatively depleted subgroup. This remains to be established.

Global DNA hypomethylation is considered to be an important epigenetic event in the development of several human neoplasms (Esteller and Herman, 2002). Folate deficiency has been hypothesised to increase cancer risk by reducing the availability of SAM for methylation of specific cytosine residues in DNA thereby permitting inappropriate proto-oncogene expression and subsequent malignant transformation. Folate deficiency in rats has been shown to decrease SAM in several tissues, but with little effect on DNA methylation (Sohn *et al*, 2003). Conversely, folate supplementation has been reported to decrease global DNA hypomethylation in seven of 20 patients with resected colonic adenomas (Cravo *et al*, 1998). In our healthy population, folate supplementation increased whole blood SAM but did not alter lymphocyte global DNA methylation. It would have been preferable to have determined the SAM : SAH ratio, as this has been reported to be more strongly related to DNA methylation than SAM alone (Sibani *et al*, 2002), but this was not possible using the method available to us at the time. Two previous studies suggested that folate depletion of healthy women leads to lymphocyte global DNA hypomethylation (Jacob *et al*, 1998; Rampersaud *et al*, 2000). We have examined responses to increasing folate intakes in healthy men and women with intakes above current daily recommendations and found no effect. This may reflect insufficient sensitivity of the method employed but equally may reflect the fact that global DNA methylation in a healthy population may be more sensitive to folate depletion than to supplementation.

Although volunteers were not recruited on the basis of genotype, and the study was not powered to identify differences in outcomes according to genotype (only seven subjects homozygous for MTHFR C677T) we did explore this possibility. MTHFR genotype had no influence on any measure of DNA stability or DNA methylation status in this study, although the authors emphasise the fact that numbers carrying the TT variant were relatively small.

In summary, supplementation with 1.2 mg folic acid in a healthy population resulted in a significant increase in several indices of blood folate status with a concomitant decrease in misincorporation of uracil into cellular DNA, particularly in the lowest quartile of RCF at baseline. DNA strand breakage, global DNA methylation status and DNA repair capacity in response to oxidative DNA damage remained unchanged in the supplemented group as a whole, although in those subjects with the lowest RCF initially, supplementation decreased DNA incision repair. The mechanism behind this observation remains to be established.

Uracil misincorporation is evidently more sensitive to improved folate status in healthy individuals than other putative biomarkers of DNA damage or repair and may, therefore, be considered a valid and functional biomarker for the influence of folate on genomic stability in healthy people.

## Figures and Tables

**Table 1:** Pre- and Postintervention biochemical variables

		**Intervention**	
**Variable**	**Group**	**Pre**	**Post**
Whole blood SAM (*μ*mol/l)	Folate	0.03 (0.03–1.04)	0.85 *(0.03–2.3)
	Placebo	0.07 (0.03–1.26)	0.07 (0.03–0.88)
			
Uracil misincorporation (arbitrary units)	Folate	51 (41–60)	40 ** (30–46)
	Placebo	47 (40.0–57.3)	42.5 (33.5–56.5)
			
Strand breaks (arbitrary units)	Folate	80 (68–96)	92 (71–103)
	Placebo	88 (71–99)	85 (68.3–107)
			
DNA incisions (BER-arbitrary units)	Folate	63 (34–85)	52 (31–81)
	Placebo	63 (40–93)	76 (53–93)
			
DNA methylation (incorporated/0.5 *μ*g DNA)	Folate	17508 (10631–19892)	15099 (12514–20121)
	Placebo	16099 (13512–18717)	17942 (14849–20732)

Median (interquartile range),

* *P*<0.01,

** *P*<0.05 response to intervention significantly different from placebo group.
